# Differential Expression of *In Vivo* and *In Vitro* Protein Profile of Outer Membrane of *Acidovorax avenae* Subsp. *avenae*

**DOI:** 10.1371/journal.pone.0049657

**Published:** 2012-11-15

**Authors:** Muhammad Ibrahim, Yu Shi, Hui Qiu, Bin Li, Amara Jabeen, Liping Li, He Liu, Michael Kube, Guanlin Xie, Yanli Wang, Guochang Sun

**Affiliations:** 1 State Key Laboratory of Rice Biology, Institute of Biotechnology, Zhejiang University, Hangzhou, China; 2 Institute of Bioinformatics, Zhejiang University, Hangzhou, China; 3 State Key Laboratory Breeding Base for Zhejiang Sustainable Pest and Disease Control, Institute of Plant Protection and Microbiology, Zhejiang Academy of Agricultural Sciences, Hangzhou, China; 4 Department of Biosciences, COMSATS Institute of Information Technology, Sahiwal, Pakistan; 5 Faculty of Agriculture and Horticulture, Humboldt-Universität zu Berlin, Berlin, Germany; Rockefeller University, United States of America

## Abstract

Outer membrane (OM) proteins play a significant role in bacterial pathogenesis. In this work, we examined and compared the expression of the OM proteins of the rice pathogen *Acidovorax avenae* subsp. *avenae* strain RS-1, a Gram-negative bacterium, both in an *in vitro* culture medium and *in vivo* rice plants. Global proteomic profiling of *A. avenae* subsp. *avenae* strain RS-1 comparing *in vivo* and *in vitro* conditions revealed the differential expression of proteins affecting the survival and pathogenicity of the rice pathogen in host plants. The shotgun proteomics analysis of OM proteins resulted in the identification of 97 proteins *in vitro* and 62 proteins *in vivo* by mass spectrometry. Among these OM proteins, there is a high number of porins, TonB-dependent receptors, lipoproteins of the NodT family, ABC transporters, flagellins, and proteins of unknown function expressed under both conditions. However, the major proteins such as phospholipase and OmpA domain containing proteins were expressed *in vitro,* while the proteins such as the surface anchored protein F, ATP-dependent Clp protease, OmpA and MotB domain containing proteins were expressed *in vivo*. This may indicate that these *in vivo* OM proteins have roles in the pathogenicity of *A. avenae* subsp. *avenae* strain RS-1. In addition, the LC-MS/MS identification of OmpA and MotB validated the *in silico* prediction of the existance of Type VI secretion system core components. To the best of our knowledge, this is the first study to reveal the *in vitro* and *in vivo* protein profiles, in combination with LC-MS/MS mass spectra, *in silico* OM proteome and *in silico* genome wide analysis, of pathogenicity or plant host required proteins of a plant pathogenic bacterium.

## Introduction

*Acidovorax avenae* subsp. *avenae* is a phytobacterium that can cause diseases in many economically important plants, including rice, corn, oats, sugarcane, millet, and foxtail [1]. In particular, rice strains of *A. avenae* subsp. *avenae* have caused severe losses in rice throughout many countries in Asia, Africa, the Americas and Europe [1], [2], [3]. The whole genome of *A. avenae* subsp. *avenae* strain RS-1 from rice has been fully sequenced in our previous study [1], which is a useful resource to identify genes involved in some specific biological functions such as the interaction between the pathogen *A. avenae* subsp. *avenae* and its rice host.

The interaction of bacterial pathogens with host cells is closely related to the expression of OM proteins, which are structurally distinct from those that reside in the inner membrane [4]. Indeed, some OM proteins from different Gram-negative bacteria not only have been recognized as important virulence factors and targets for host immune recognition [5], but also have recently been proposed to be required for the bacterial Type VI secretion system (T6SS). This represents a new paradigm of protein secretion that is critical for the pathogenesis of many Gram-negative bacteria [6].

Identification of abundant and/or novel OM proteins, in particular OM components of the T6SS, and characterization of their roles in pathogen physiology, disease, and defense against the host, is an important preliminary step in understanding the pathogenesis [5]. Recently, a variety of studies have investigated the OM proteome of Gram-negative bacteria including *Xanthomonas campestris*, *E. coli*, *Aeromonas salmonicida*, *Proteus mirabilis*, *Burkholderia pseudomallei*, *Dickeya dadantii*, *Burkholderia mallei*, *Yersinia pestis, Actinobacillus pleuropneumoniae* and *Bartonella henselae* [5], [7], [8], [9], [10].

Most of this research focused on the proteome of *in vitro* cultivated bacteria [11]. However, it is the characterization of the bacterial OM proteome and T6SS during *in vivo* infection of its host that eventually could provide the most significant insights into bacterial pathogenesis [12]. Indeed, proteomic analysis of OM proteins in human and animal pathogens such as *Proteus mirabilis* has revealed differential expression *in vivo* vs. *in vitro* conditions. In contrast, little is known about the *in vivo* expression profile of plant pathogenic bacteria due to the absence of an efficient method to obtain bacterial cells from plant tissue.

In this study, we developed a method to collect the *in vivo* bacteria from rice plants, which makes it possible to analyze *in vivo* OM proteome and the T6SS of *A. avenae* subsp. *avenae* strain RS-1. We present the *in vitro* vs. *in vivo* OM proteome profiling by using LC-MS/MS in combination with an *in silico* analysis of OM proteome and T6SS proteins of *A. avenae* subsp. *avenae* strain RS-1, while proteomic analysis of OM proteins revealed the differential composition between *in vivo* and *in vitro*. In particular, T6SS core components OmpA/MotB domain containing proteins and an ATP dependent Clp protease was identified in the OM proteome under *in vivo* conditions, but not under *in vitro* conditions. This validated the *in silico* prediction of the existance of T6SS core components or associated proteins and highlighted that several OM proteins may be involved in the survival and pathogenicity of *A. avenae* subsp. *avenae* strain RS-1 in host plants.

## Results and Discussion

In this study, we applied a strategy combining the benefits of LC-MS/MS for a comprehensive coverage of proteins from host recovered bacteria with *in silico* predicted OM proteins and genome wide predicted T6SS proteins. Results of this study clearly revealed the differential expression of OM proteins in *A. avenae* subsp. *avenae* strain RS-1 between *in vivo* and *in vitro*. Indeed, a non-redundant list of OM proteins has been assembled in *A. avenae* subsp. *avenae* strain RS-1 by using LC-MS/MS and *in silico* prediction (Tables S1, S2). Of the proteins, 48 were identified as shared proteins under both *in vitro* and *in vivo* conditions (Table S3). However, 49 proteins were identified under *in vitro* but not *in vivo* (Table S4), while 14 proteins identified under *in vivo* but not *in vitro* (Table 1). Overall, the results confirmed our main hypothesis that the expression of OM proteins may play key roles in the interaction of bacterial pathogens with host cells and survival within the host cells.

**Table 1:** List of LC-MS/MS identified unique outer membrane protiens under *in vivo* condition of *Acidovorax avenae* subsp. *avenae* strain RS-1 proteome.

Locus Tag	Unique outer membrane protiens *in vivo*
Acav_0812	TonB-dependent receptor/*A. avenae* subsp. *avenae* ATCC 19860
Aave_3310	Binding domain containing prottein/*Acidovorax citrulli* AAC00-1
Aave_2079	unnamed protein product/*Acidovorax citrulli* AAC00-1
Aave_4400	phasin family protein/Acidovorax avenae subsp. avenae ATCC 19860
Aave_3309	flagellin/*Acidovorax citrulli* AAC00-1
Acav_0496	OmpA/MotB domain-containing protein/*A. avenae* subsp. *avenae* ATCC 19860
Acav_2010	ATP-dependent Clp protease/*A. avenae* subsp. *avenae* ATCC 19860
Acav_3880	General secretion pathway protein F
Acav_4244	porin Gram-negative type/*A. avenae* subsp. *avenae* ATCC 19860
Acav_4297	flagellin domain-containing protein/*A. avenae* subsp. *avenae* ATCC 19860
Acav_4298	flagellin/*A. avenae* subsp. *avenae* ATCC 19860
AcdelDRAFT_1224	Phosphate ABC transporter PBP/*Acidovorax delafieldii* 2AN
Ajs_0682	porin Gram-negative type/*Acidovorax* sp. JS42
	Major OM lipoprotein/*Pseudomonas oleovorans*

### *In vivo* vs. *in vitro* 1D Profile of OM Proteins

**Figure 1:**
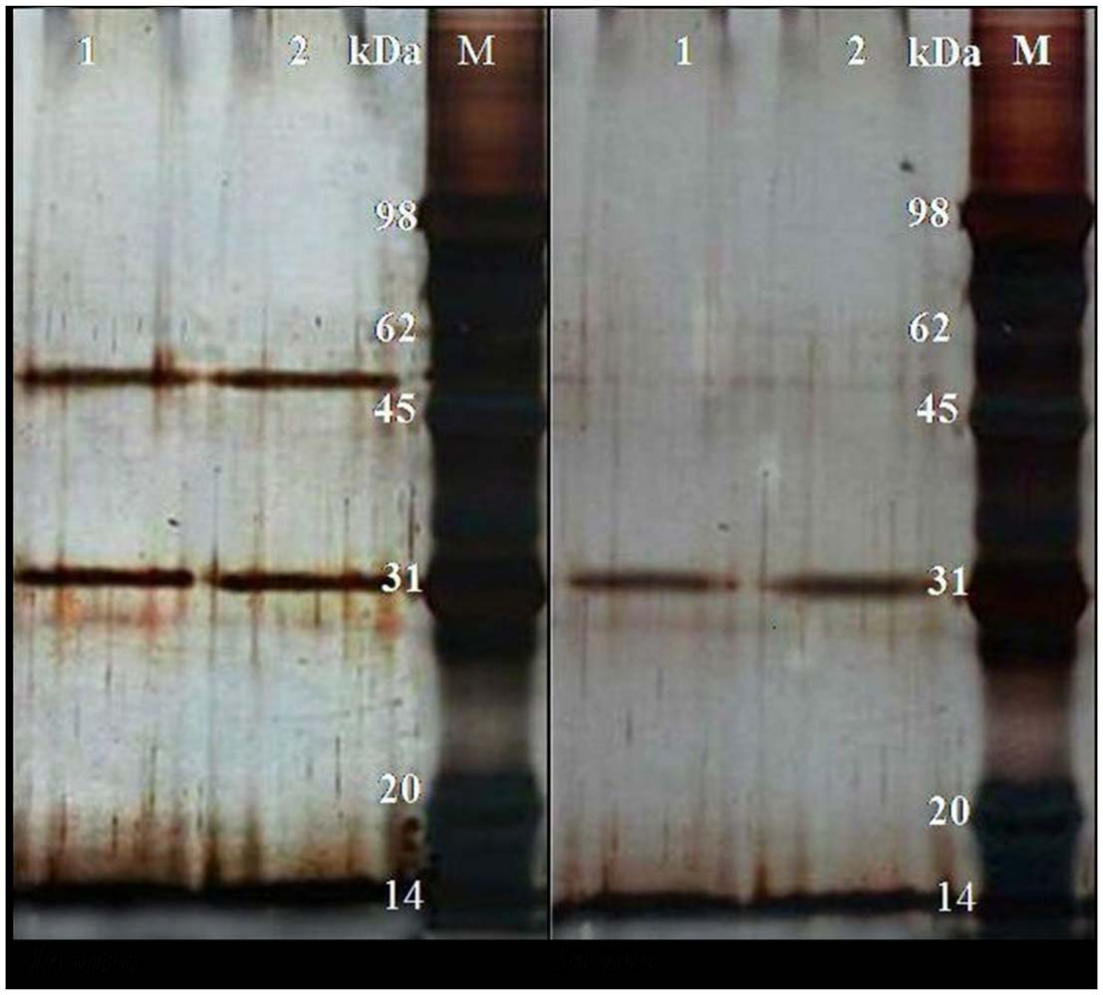
SDS-PAGE profile of *Acidovorax avenae* subsp. *avenae* strain RS-1 under *in vivo* and *in vitro* conditions. A) Represents the *in vitro* growth conditions where Lane M represents the marker while Lane 1 and 2 represent the two replicates. B) Represents the *in vivo* growth conditions where Lane M represents the marker while Lane 1 and 2 represent the two replicates.

The *in vitro* and *in vivo* OM proteins of *A. avenae* subsp. *avenae* strain RS-1 were prepared as described in Materials and Methods and proteins were separated by 1D SDS-PAGE. As shown in Figure 1, the OM protein profiles of *A. avenae* subsp. *avenae* strain RS-1 *in vivo* are different from those of *in vitro* in general. Although, there is a high similarity in some protein bands between *in vitro* and *in vivo*. The protein bands that appeared by silver staining were cut from the gel into small pieces and were further digested with trypsin. The resultant peptides were then analyzed with the help of LC-MS/MS.

### Global Proteins Profiling of RS-1 Proteome

LC-MS/MS profiles of the peptides from *A. avenae* subsp. *avenae* strain RS-1 were used for searching the sequence similarities to data from *A. avenae* or closely related Gram-negative bacteria available in the NCBI database. A proteome-wide dataset of proteins was compiled for putative protein localization in *A. avenae* subsp. *avenae* strain RS-1. Two subcellular localization predictor (PSORTb and Phobius) were used to assemble an inventory of potential OM proteins encoded in the genome of *A. avenae* subsp. *avenae* strain RS-1 and identified by LC-MS/MS. Of the total 5,043 proteins of *A. avenae* subsp. *avenae* strain RS-1 submitted to PSORTb and Phobius [13], [14], 85 were predicted to be localized to the OM. In-gel tryptic digestion data (LC-MS/MS) from two biological replicates were in general consistent, and the combined data from two biological replicates revealed more than 97 putative OM proteins with>98% confidence *in vitro*, while more than 62 putative OM proteins with>97% confidence *in vivo*. After retrieving proteins identified under *in vitro* and *in vivo* condition and removing the overlapping protein entries and cytoplasmic proteins from each conditions, a non-redundant list of 159 proteins (*in vitro* and *in vivo*) was assembled and designated as OM as well as probable OM proteins in *A. avenae* subsp. *avenae* strain RS-1 by PSORTb and Phobius (Tables S1, S2). Of the 97 putative OM proteins under *in vitro* conditions, 63 proteins were among the list of 85 OM proteins predicted by PSORTb and Phobius [13], [14] and similarly, of the 62 putative proteins under *in vivo* conditions, 45 were among the list of 85 OM proteins predicted by PSORTb and Phobius [13], [14] in the genome of *A. avenae* subsp. *avenae* strain RS-1.

PSORTb and Phobius analysis [13], [14] of the above identified 159 OM and probable OM proteins showed that most of these proteins are homologous to well-documented OM associated proteins, such as TonB-dependent receptor, OM efflux protein, OmpA domain-containing protein and porin Gram-negative type, with high and medium confidence levels (Tables S1, S2). However, about 5% of all OM proteins, such as the LysR family transcriptional regulator, the gamma subunit of the F0F_1_ ATP synthase, a phasin family protein and several so far uncharacterized proteins, were predicted to have multiple localization sites based on a statistical analysis of the SwissProt database, Phobius [13], [15] and PSORTb [14], and were included in the list of lipoproteins and other probable OM proteins (Tables S1, S2).

### Elucidation and Characterization of *in vitro* OM Proteome

In total, 97 proteins were identified in the OM and probable OM proteome of *A. avenae* subsp. *avenae* strain RS-1 by LC-MS/MS under *in vitro* conditions. After retrieving proteins identified under *in vitro* conditions and removing the overlapping protein entries, a non-redundant list of 63 OM proteins remained from the whole list of OM proteins that had been predicted based on *in silico* analysis of *A. avenae* subsp. *avenae* strain RS-1 by PSORTb and Phobius (Table S1). Almost all of the 63 OM proteins identified in strain RS-1 of *A. avenae* subsp. *avenae* had the highest sequence similarity with those of *A. avenae* subsp. *avenae* strain ATCC19860 with the exception of a few proteins showing the highest level of sequence similarity to other plant pathogens (Table S1). The detailed topology of LC-MS/MS identified OM proteins in *A. avenae* subsp. *avenae* strain RS-1 is as follows:

Twelve TonB-dependent receptor/complex proteins (TBDR) (COG1629) were identified in the OM of *A. avenae* subsp. *avenae* strain RS-1 (Table S1). This result is consistent with the result of Blanvillain *et al*. [16], who found that the OM of Gram-negative bacteria is enriched with TBDR, a family of beta-barrel proteins. However, the number of TBDRs in this study is about twice that of *B. pseudomallei* [5], which may be attributed to the difference in bacterial species. The TonB complex works to sense signals from outside the bacterial cell and transmits them via two membranes into the cytoplasm, leading to transcriptional activation of target genes [17]. TBDRs are also carbohydrate scavenger [16] and may play a key role in sensing and exploitation of plant derived carbon sources for *A. avenae* subsp. *avenae*. Furthermore, TonB is often involved in metal acquisition, especially iron, which is essential for all bacteria [17], and it also has been reported as an amylosucrase and a regulator of sucrose utilization [16].

Eleven proteins have been assigned as porins (COG3203), and have been presented in Table S1. They comprised the second populous group of OM proteins in *A*. *avenae* subsp. *avenae* strain RS-1 proteome. This result is consistent with previous studies that demonstrated that Gram-negative bacteria encode in high number porins [5], [10], [18], [19], [20]. Porins are beta barrel folded proteins that provide water-filled channels through which molecules can diffuse and allow the diffusion of hydrophilic molecules across the OM. They also can act as receptors for bacteriophages and are involved in a variety of functions including principal molecular determinant of phage susceptibility [21]. We have identified 5 OM efflux proteins (COG1538), which is a group of lipoproteins from the NodT family of the RND (Resistance-Nodulation-cell Division) type efflux systems that form trimeric channels with a β-barrel to allow export of diverse substrates including antibiotics [17], [22]. These efflux proteins work with an inner membrane ABC transporter protein (ATPase subunit) and an adapter called a membrane fusion protein. Most of these efflux proteins are likely to export primarily small molecules rather than proteins and are related to the type I secretion OM proteins TolC and PrtF. The presence of NodT family of RND efflux pump in *A*. *avenae* subsp. *avenae* strain RS-1 may thus be functionally similar to those in *E. coli* and other Gram-negative bacteria, where they act as a major efflux system involved in the resistance of bacteria to a variety of toxic molecules, including antibiotics, dyes and detergents [18]. In addition, two members of the OmpW and OmpA families were also identified in the OM proteome of *A*. *avenae* subsp. *avenae* strain RS-1. These proteins are believed to play multifunctional roles but the mode of action remains unclear [5].

We also have identified the flagellin N-terminal helical region (COG1344) which includes flagellin and hook associated protein (Aave_4400, XAC1975, Aave_4400, Acav_4298, Acav_4297, Aave_4401) and are reported to form extended helix structures [23]. The HflC (Aave_1431) and HflK (Aave_1432) proteins were assigned to the COG0330 domain, which is the N terminal of the bacterial membrane proteins. HflK complexes with HflC to form a membrane protease that is modulated by the GTPase HflX [24]. In addition, these proteins are well understood as hypersensitive induced reaction (HIR) proteins and along with prohibitins and stomatins form a PID (proliferation, ion and death) proteins superfamily. These PID proteins share common features in their SPFH (stomatins, prohibitins, flotillins, HflK/C) domains [25]. Toluene tolerance protein (COG2850), which is mediated by an increase in cell membrane rigidity, was also identified. It is a transporter and shows similarity to the ABC transporter. The RHS protein (COG3209), which contains extended repeat regions that often appear to be involved in ligand binding [26], methyl accepting chemotaxis sensory transducer (COG0840), 3 extracellular solute binding proteins (PFLU1139, Aave_4073, Acav_3681) that was assigned to COG0834, and surface antigen proteins (COG0729) (Table S1). Moreover, 3 general secretary pathway proteins (COG1450), are predicted to be a pore for type III and type IV secretion system used to export proteins across the OM (Table S1).

Thirteen unnamed proteins were also identified in the OM of *A. avenae* subsp. *avenae* strain RS-1. These proteins were assigned to families and domain entries of the Pfam database [27]. Acav_1013 and Acav_2514 were assigned to COG0457, while Aave_1831 belongs to TPR (Tetratricopeptide-like repeats) family proteins (COG4775), which are reported to be involved in the functions such as cell cycle regulation, transcriptional control, mitochondrial and peroxisomal protein transport, neurogenesis and secretion systems [28], [29]. Similar to *Ralstonia solanacearum*, TPR proteins in *A. avenae* subsp. *avenae* strain RS-1 may act as type-III-secretion regulators and chaperones [29]. The unnamed protein (Aave_0155), a family of OmpW in COG3047, is known as a family of related proteins, such as porin-like integral membrane proteins [30]. Acav_2307 has no known COG and in Pfam analysis the sequence was notably matched with the integral membrane proteins or lipid anchored proteins of the OmpA family [31]. The unnamed protein with locus tag Aave_4344 was identified as a porin (COG3203) belonging to the general bacterial porin family and acts as a molecular filter for hydrophilic compounds [32]. The unnamed protein Aave_1519 was identified as a universal stress protein (COG0589); Acav_3854, Acav_2307, Acav_4055, and Acav_0287 were assigned to a protein family of unknown function. No COG or Pfam assignment was possible but the deduced proteins carry signal peptides (*in silico* data not shown). These likely represent examples of new types of OM proteins with functions that are yet to be elucidated.

Of the 63 identified OM proteins, 16 proteins possessed a GRAVY -value>0, indicating that some hydrophobic proteins were also identified by this approach [33], including LysR family transcriptional regulator, lipoproteins and an OmpW protein family member (Table S1). A previous study indicated that Gram-negative bacteria have hydrophilic OMs, which act as a barrier against hydrophobic molecules and require transporters to facilitate diffusion into the cytoplasm [34]. Here, we suggest that NodT and OmpW may function as a transporter to facilitate their diffusion into the cells of *A. avenae* subsp. *avenae* strain RS-1.

In general, in agreement with the result of Tjalsma *et al*. [35] and Schell *et al*. [5], our results also revealed the identification of peptides that were recognized as cytoplasmic proteins and cytoplasmic associated proteins, for example, ribosomal proteins, glyoxalase resistance protein/dioxygenase, DNA topoisomerase, elongation factor, tetraacyl disaccharide, ATP synthase subunit beta, Dead/Death box helicase domain-containing, chaperonin GroEL, phosphoglucomutase, ribonuclease E, ribonuclease rng/rng family, 3-isopropylmalate dehydratase, gluconolactonase and pyruvate kinase. However, most of the proteins in this study identified by LC-MS/MS were OM proteins, lipoproteins and probable OM proteins (Table S1).

In this comprehensive list of *A. avenae* subsp. *avenae* strain RS-1 OM proteins and probable OM proteins, many are indispensible for the survival and growth of *A. avenae* subsp. *avenae* strain RS-1 and accessible on the surface of *A. avenae* subsp. *avenae* strain RS-1. Therefore, it could be suggested that the depletion of them either could leads to rapid accumulation of unassembled OM proteins in the periplasm followed by cell death as in *E. coli* [33].

### Elucidation and Characterization of *in vivo* OM Proteome

As shown in Figure 1, a marked difference was observed in the 1D profile of OM proteins in *A. avenae* subsp. *avenae* strain RS-1 between *in vitro* and *in vivo*. For LC-MS/MS analysis, peptides released from the *in vivo* expressed OM proteome were pooled from two biological replicates. After removing redundant data, the *in vivo* data were compared with *in vitro* data, and this indicated that the *in vivo* expressed OM proteome is different from that found *in vitro* (Tables S1, S2). Altogether, 62 proteins were obtained *in vivo* after the comprehensive analysis of two replicates (Table S2), while most of them were lipoproteins, porins, NodT dependent receptors, and TDBR. In addition, in agreement with the result of *in vitro* experiment, some probable OM proteins like flagellin domain proteins, ABC transporter subunits were also identified *in vivo*. However, about 7% of all proteins were also detected as cytoplasmic proteins. The majority of these proteins were assigned as ribosomal proteins, DNA topoisomerases and elongation factors.

Overall, results from *in vivo* study indicated that porin (COG3203), a major OM proteins were generally more populous *in vivo* than *in vitro*, which may support the report that porin may play a pivotal role in the bacterial survival in the environment [36]. Therefore, under *in vivo* conditions, it might be expected that the relative proportion of porins would increase so that the cell could scavenge more successfully for traces of sugars and other carbon sources. Large number of differentially expressed proteins was identified in the OM from strain RS-1 of *A. avenae* subsp. *avenae* in *in vivo* compared to *in vitro.* Particularly, F proteins (COG1459) which form a platform for the machinery of the type II secretion system (T2SS), as well as the type IV pili and proteins similar to archaeal flagella [37] were not identified *in vitro* but were identified *in vivo* (Table 1). In Gram-negative bacteria, there is evidence that T2SS secretes several toxins across the OM [37] and most of the Type II secreted proteins that have been characterized to date are involved in degrading different components of plant cell walls. Therefore, results presented here strongly support this hypothesis that the F proteins may be involved in the pathogenicity of rice *A. avenae* subsp. *avenae* pathogen.

Proteins of COG1360 contain an OmpA/MotB domain and are thought to function as porin-like integral membrane proteins or lipid-anchored proteins [38] were identified *in vivo* (Table 1). The flagellar motor protein MotB and the outer membrane protein OmpA share a region of sequence homology, which describes a domain found fused to T6SS homologues of the T6SS protein DotU, with OmpA/MotB homology. Furthermore, ATP-dependent Clp protease (COG0542) was identified only in *in* *vivo* condition (Table 1). It belongs to the AAA+super family of ring-shaped P-loop NTPases and was associated with diverse cellular activities [39]. Interestingly, a three dimensional fold increase of ClpB matched with the folds of ATP-dependent Clp protease (ClpA) and to chaperone Hsp104 and related ATP-dependent Clp proteases, which belong to T6SS [40].

### Functional Differential Expression of the *in vivo* and *in vitro* Proteome

It was established from the LC-MS/MS data that most of proteins were decreased under *in vivo* conditions compared to *in vitro* conditions (Table S2), although some unique proteins only expressed under *in vivo* conditions. The abundance changes of these proteins are provided in (Tables 1, S4). Most of the TonB, NodT and ABC transporter proteins were decreased, indicating that these proteins may be less active under *in vivo* conditions, but some TonB and ABC transporter proteins, which were also identified but have different sequence homologies compared to *in vitro*. The family of TonB uses S-adenosylmethionine in the methylation of diverse substrates and the member of this family includes a related group of bacterial proteins of unknown function domains belonging to the periplasmic binding protein superfamily ABC transporter proteins. In addition, a remarkably high number of TDBR in strain RS-1 of *A. avenae* subsp. *avenae* under *in vitro* compared to *in viv*o is inconsistent with the result of Tsolis *et al*. [41], who found that TonB dependant proteins contribute to the growth of bacterium in the host (mouse) and indicate the complex functions of these proteins. This could be due to difference of bacterial species and host.

From the lists of OM proteins identified under both *in vitro* and *in vivo* conditions, we derived a catalog of shared OM proteins irrespective of growth conditions. The 48 most abundant of these shared OM proteins are listed in Table S3 and represent about 40% of total OM proteins in the *A. avenae* subsp. *avenae* strain RS-1. Among these shared proteins, the vast majority are porins, TDBR, ABC transporter proteins, flagellin and OmpW-like proteins. The fact that these are abundantly present in cells growing in different growth conditions implies that these shared proteins are fundamental to physiology of *A. avenae* subsp. *avenae* regardless of the growth conditions. Therefore, these proteins are suggested to represent key elements in controlling detrimental aspects of this bacterium to rice plant, and to exploit the beneficial aspects of this bacterium in plant health.

However, the presence of Protein F, ATP-dependent Clp proteases and OmpA/MotB, as well as some unique flagellin (limited to *in vivo* condition; Table 1), indicate their importance in bacterial niche adaptation and virulence. Protein F, a component of the type II secretion system (T2SS), is believed to form a membrane complex and serves as a platform on which the pseudopilus is built [42]. The T2SS is a multimeric protein complex and has been reported for pathogenic Gram-negative bacteria to secrete virulence determinants which are commonly toxins and invading the host cells [43]. ATP-dependent proteases have been well understood for their association with T6SS and pathogenicity in Gram-negative bacteria [7]. ATPases are frequently used by secretion systems to energize the transport process, while in the case of the T6SS, a gene encoding ClpB homologue is more frequently found [44]. This was confirmed by a genome wide analysis of *A. avenae* subsp. *avenae* strain RS-1 in our current study (next section). OmpA is one of the immunodominant antigens and binding of specific anti-OmpA antibodies leads to cell lysis in the presence of complement and can interact with host receptor molecules [45]. MotB (and MotA) serve two functions, the MotA/MotB complex attaches to the cell wall via MotB to form the stator of the flagellar motor, and the MotA/MotB complex couples the flow of ions across the cell membrane to movement of the rotor in most of the Gram-negative bacteria [38].

The association of OmpA/MotB and ATP-dependent Clp proteases with the T6SS make it possible that T6SS may play an important role in survival and pathogencity of *A. avenae* subsp. *avenae* strain RS-1. Indeed, the T6SS has been identified in>80 genomes of Gram-negative bacteria, accounting for about 25% of currently available bacterial genome sequences. In addition, recent studies have shown that the T6SS has been linked to a variety of functions such as bacterial pathogenicity, host adherence and colonization, cytotoxicity, host-cell invasion, biofilm formation, conjugation, quorum-sensing regulation, survival within macrophages, persistence within the host, both promoting and limiting virulence [46].

### Genome Wide Analysis of T6SS Proteins

**Figure 2:**
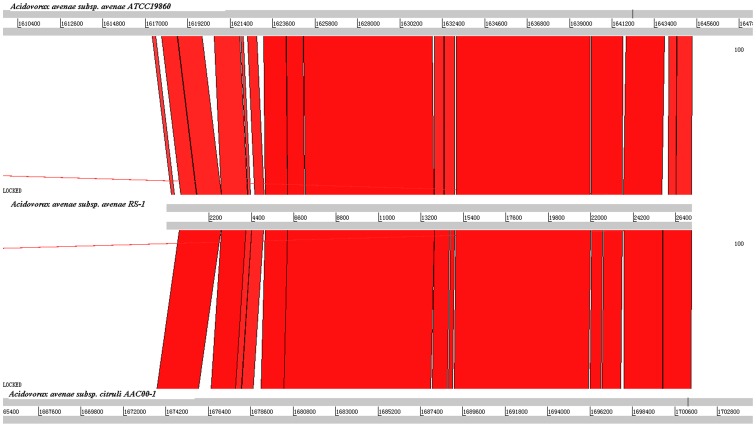
Comparison of the T6SS locus of *Acidovorax avenae* subsp. *avenae* strain RS-1 and closely related bacteria by TBLASTN analysis using WebACT and visualized with ACT software.

It is well known that many bacteria used specialized secretion systems to inject DNA or proteins into organisms like human, animals or plants [47]. However, little is known about the components, location and molecular mechanism of T6SS in bacteria, particularly, plant pathogenic bacteria. Currently, several studies have reported the association of several T6SSs with inner or outer membranes [5], [7]. Thus, a genome wide analysis was performed in this study to investigate the association of OM proteins with T6SS. Using 13 T6SS core components described by Boyer *et al*. [47] as bait sequences, local BLAST (BLASTN, BLASTX) were used to examine the T6SS locus of *A. avenae* subsp. *avenae* strain RS-1. T6SS loci were located on three contigs (AAARScontigs 66, AAARScontigs 67 and AAARScontigs 68). For DNA based comparison, three contigs of *A. avenae* subsp. *avenae* strain RS-1 containing the T6SS were fused and compared to *A. avenae* subsp. *citrulli* AACOO1 and *A. avenae* subsp. *avenae* ATCC19860 using WebAct [48] and visualized by ACT [49] (Fig. 2).

**Figure 3:**
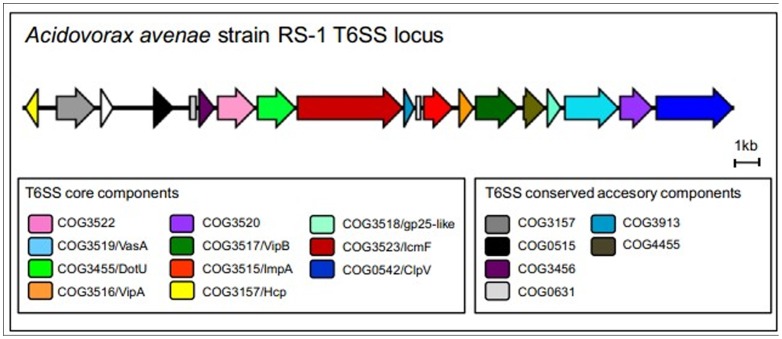
Locus of T6SS core and conserved accessory components in *Acidovorax avenae* subsp. *avenae* strain RS-1 which also exist in *Acidovorax avenae* subsp. *citrulli* strain AAC00-1 or *Burkholderia thailandensis* strain E264 are represented with a different color.

In this study, analysis revealed that only one T6SS gene cluster was found in strain RS-1 of *A. avenae* subsp. *avenae* encodes T6SS core and conserved accessory components (Fig. 3 and Table S5). As compared to the T6SS of the *A. avenae* subsp. *citrulli*, the T6SS locus in *A. avenae* subsp. *avenae* does not encode either a VgrG homolog or additional T6SS gene cluster, indicating that this T6SS locus encoded single gene cluster. In addition, bioinformatics analysis of genomic sequence revealed the presence of 12 ORFs encoding putative VgrG proteins as an orphan component in *A. avenae* subsp. *avenae* strain RS-1 (Table 2). However, except OmpA/MotB and ClpB, no T6SS protein, even VgrG, was confirmed from LC-MS/MS analysis. One may speculate, if this is the due to their location on the inner membrane or in periplasm.

**Table 2:** Presence of VgrG homologous as an orphan components in *Acidovorax avenae* subsp. *avenae* strain RS-1.

Serial No.	VgrG homologs (COG3501)	KEGG annotation
1	Aave_0241	Rhs element Vgr protein
2	Aave_0481	Rhs element Vgr protein
3	Aave_0497	Rhs element Vgr protein
4	Aave_2047	Rhs element Vgr protein
5	Aave_2127	Rhs element Vgr protein
6	Aave_2735	Rhs element Vgr protein
7	Aave_2840	Rhs element Vgr protein
8	Aave_3347	Rhs element Vgr protein
9	Aave_3486	Rhs element Vgr protein
10	Aave_3752	Rhs element Vgr protein
11	Aave_3783	Rhs element Vgr protein

Interestingly, a genome wide sequence analysis of various bacterial species and strains suggests that the avirulent species often lack a T6SS, whereas pathogenic species often encode orthologs of T6SS as shown for animal pathogens and phytopathogenic bacteria such as *Xanthomonas populi* and *Xanthomonas codiaii* [40]. However, orthologs of four hypothetical proteins VCA0118, VCA0119, VCA0121 and VCA0122 were not identified in *A. avenae* subsp. *avenae* strain RS-1. This is in agreement with the result of Shrivastava and Mande [40]. They showed that bacterial species such as *Burkholderia* and *Xanthomonas* lacking one or more of the T6SS orthologs mostly lack orthologs of VCA0118, VCA0119, VCA0121 and VCA0122. This finding indicates that these proteins may be related to T6SS.

Phylogenetic analysis of sequences in this study clearly revealed the association of the predicted T6SS proteins in *A. avenae* subsp. *avenae* strain RS-1 with that of the closely related bacteria such as *A. avenae* subsp. *citrulli* strain AAC00-1 and *A. avenae* subsp. *avenae* ATCC19860 (Fig. S1).

### Conclusions

In this study, a Triton-insoluble fraction of lysozyme-treated *A. avenae* subsp. *avenae* strain RS-1 was digested with RNase yielding the purified OM preparation from *in vitro* and *in vivo* condition*s.* Trypsin shaving of *A. avenae* subsp. *avenae* strain RS-1 OM preparations from *in vitro* and *in vivo* growth by LC−MS/MS identified>159 OM proteins and probable OM proteins, while *in silico* functional analysis also predicted their function as OM or OM-associated (e.g., biosynthesis of EPS, LPS, and cell wall, T6SS, cell division, motility etc). This result revealed that the *in vitro* vs. *in vivo* approach developed in this study may also be applied to proteomic comparative analysis of other plant pathogenic bacteria. Furthermore, the comparative global proteomic survey of *A. avenae* subsp. *avenae* strain RS-1 grown *in vitro* vs. *in vivo* revealed differential expression of proteins geared towards survival and pathogenicity of the rice pathogen in host plants. The most dominant proteins induced under *in vivo* conditions are the secretary proteins, the machinery of the T2SS, type IV pili, T6SS, and the archaeal flagella. In particular, the LC/MS identification of OmpA/MotB and ClpB, validated *in silico* prediction of T6SS core components based on the sequenced genome of *A. avenae* subsp. *avenae* strain RS-1. Overall, our *in silico* functional characterization have most probably confirmed the identified OM proteins and probable OM proteins based on *in vitro* and *in vivo* LC/MS analyses of proteins in *A. avenae* subsp. *avenae* strain RS-1. Even though the structure and topology of these machines in depth remain to be fully elucidated. In summary, we made several novel observations in the probable OM localization of T6SS components in particular OmpA/MotB, while several proteins like protein F, T2SS, porins, OmpA, ATP dependent protease and ABC transporter proteins may be required for the survival and pathogenicity of *A. avenae* subsp. *avenae* in host plants.

## Materials and Methods

### Bacterial Growth and Culture Conditions

Strain RS-1 of *A. avenae* subsp. *avenae* was isolated from rice plants in China [1], [3] and stored in 20–30% sterile glycerol (Shanglin Industries, Hangzhou, China) at −80**°**C. For *in vitro* analysis, the bacterial culture was maintained on fresh Luria Bertani (LB) media plates and incubated at 30**°**C for 24 h. Single colonies were inoculated into 10 mL of LB broth and incubated at 30°C for overnight. The overnight bacterial cells were re-cultured into 20 mL fresh LB broth for 6–8 h from initial cell densities of 0.1 O.D_600nm_ until harvest at late exponential or early stationary phase (O.D_600nm_ between 1.8–2.0). Cells were harvested by centrifugation at 8000 rpm for 10 min and processed for OM preparation.

For *in vivo* analysis, bacterial strain was inoculated and recovered as follows. Six days after the inoculation, infected leaves were collected, decontaminated with alcohol. Leaves were cut into pieces with a sterile razor blade and maintained for 20 min in sterile glass plates containing 20 mL of distilled water. The incubation during this period of time allowed the bacteria to detach from the leaf tissues. The leaves were separated from the suspension and collected by centrifugation. The bacterial cell-pellets were washed with phosphate buffer saline (PBS) and with water and then used for protein extraction.

### Preparation of OM Proteins

Bacterial cells of *A. avenae* subsp. *avenae* strain RS-1 were collected at late-exponential or early stationary phase from LB broth (referred to as *‘in vitro’*) and from the leaves of 6 d old infected rice plants (referred to as ‘*in vivo*’), respectively. OM protein extraction was carried out as described earlier [20]. Briefly, the cells were treated with lysozyme 60 µg/mL in the membrane buffer (10 mM Tris, pH 8.0, 0.75 M sucrose, 2 mM EDTA and 1 mM PMSF), disrupted by sonification and centrifuged at 8000 rpm for 10 min at 4°C. The supernatant was recovered and centrifuged at 30,000 rpm for 2 h at 4°C. Pellets were suspended with 2% Triton X-100 in the membrane buffer and incubated at room temperature for 30 min and centrifuged at 30,000 rpm for 2 h at 4°C to isolate the inner membrane proteins. The OM proteins remained in the pellet. The OM protein enriched pellets were suspended in the lysis buffer (8.5 M urea, 1.98 M thiourea and 2% CHAPS) and separated by 1D SDS-PAGE. The purified OM enriched fraction was treated with RNase and EDTA to remove ribosomal contamination as described by Schell *et al*. [5].

### One-dimensional SDS-PAGE

Membrane proteins of *A. avenae* subsp. *avenae* strain RS-1 were separated on a 10% 1D SDS-PAGE. The samples were analyzed according to the method of Laemmli *et al*. [50] with some modification using an acryl amide concentration of 5% for the stacking gel and 12% for the running gel. SDS-PAGE was performed with a mini-gel apparatus (VE-180 vertical electrophoresis bath, Tanon, China). Separated protein bands in the SDS-PAGE gel were visualized with both silver staining and Coomassie brilliant blue.

### LC-MS/MS of Trypsin-Digested Proteins

The peptides from purified OM were prepared in two biological replicates and analyzed by LC-MS/MS. Tryptic peptides (10 µL) obtained from each band of the SDS-gel of bacterial cell lysate were separated on a Dionex Ultimate 3000 nano-LC system with a Dionex Acclaim PepMap 100, C18 trap column (20 mm × 100 µm, 5 µm, 100 Å) and Dionex Acclaim PepMap 100, C18 analytical column (150 mm×75 µm, 3 µm, 100Å). The tryptic peptides were loaded onto the trap column using 2% ACN with 0.025% TFA at the flow rate of 10 µL/min. The enriched tryptic peptides were then separated through the analytical column by gradient elution. The flow rate and column temperature were kept at 250 nL/min and 25°C, respectively. Mobile phases A (0.1% formic acid in 5% acetonitrile) and B were 0.1% formic acid in 40% acetonitrile. The gradient was started at 0% B to 90% B in 40 min. The amaZon ETD ion trap MS equipped with the nano source was used to analyze the tryptic peptides. The scan range for MS and MS/MS were set at 300–1400 m/z and 50–2200 m/z, respectively. AutoMS/MS was applied with fragmentation at amplitude of 30%–200%.

The obtained LC/MS spectra were evaluated by using the MASCOT LC-MS/MS ion search algorithm (Matrix Sciences). All the MS/MS spectra were analyzed by selecting the enzyme trypsin and applying the search parameters of precursor tolerance of 0.35 Da and a fragment tolerance of 0.35 Da, oxidation of methionine and carboxyamidomethylation of cysteine were considered as variable and fixed modification, respectively. LC-MS/MS profiles of the peptides from *A. avenae* subsp. *avenae* strain RS-1 were used for searching the sequence similarities to data from *Acidovorax* species available at NCBI. The cross correlation scores (X corr) [51] of singly-, doubly- and triply-charged peptides were greater than 1.8, 2.5 and 3.5, respectively, were fixed for protein identification. The program listed the peptides corresponding to the proteins. A list of peptide sequences that had the highest × corr values was identified. Other parameters of ΔvaCn>0.1 are selected for anticipated results in addition to Xcorr score. The proteins were identified either by sufficient number of peptides or identified at least by one peptide that is redundant enough to be considered reliable with acceptable scores. After identifying the proteins with the set threshold values, even the peptides below the defined thresholds were also considered to increase the sequence coverage. The final list of proteins was prepared by combining all the proteins obtained from different LC-MS/MS runs after manual verification. The redundancy of peptides within the list of peptides from each protein was removed by verifying both the m/z value and the corresponding sequence. The redundant proteins were also removed.

### *In silico* Analysis of LC-MS/MS Released Peptides

A genome-wide prediction of protein sub cellular localization has been performed for strain RS-1 of *A. avenae* subsp. *avenae* in our previous study [1] using PSORTb (version 3.0.2) and Phobius [13], [14] by selecting Gram-negative strains with normal format and at significant score>7.5. LC-MS/MS profiles of the peptides from *A. avenae* subsp. *avenae* strain RS-1 were used for the analysis of the sequence similarities using local BLAST, while complete protein sequences were retrieved from the genome of *A. avenae* subsp. *avenae* strain RS-1. The grand average of hydropathicity (GRAVY) score of peptides were also evaluated using the ProtParam tool of ExPASy to look at the hydrophobic nature of proteins and SignalP 3.0 server [52] was used to hypothesize N-terminal secretary signal peptides of the identified proteins. Furthermore, proteins annotated by RAST automatic pipeline were used for functional analysis, while locus tag names were assigned from the genome sequenced strain ATCC19860 of *A. avenae* subsp. *avenae* or more closely related Gram-negative plant pathogenic bacteria. In addition, a list of *A. avenae* subsp. *avenae* strain RS-1 OM-associated proteins that related with other plant pathogen was also manually assembled by using keywords such as OM, lipoprotein, flagella, pili, secretin, porin, receptor, and surface at Integrated Microbial Genomes (IMG).

### *In silico* Analysis of Type VI Secretion Loci

A genome wide analysis was carried out to get an overview of T6SS proteins in *A. avenae* subsp. *avenae* strain RS-1. Identification of T6SS homologous coding loci were conducted by BLASTN and BLASTX searches using the 13 T6SS core components as baits [47]. For comparative analysis of T6SS, nucleotide sequences were aligned by BLASTN and BLASTX, compared using WebAct online source [49] and visualized by Artemis Comparison Tool (ACT) release 6 [48]. In addition, to identify genes that were represented across *A. avenae* subsp. *avenae* strain RS-1, *Pseudomonas aeruginosa, Burkholderia mallei, A. avenae* subsp. *citrulli* strain ACC001 and *Vibrio cholerae*, a phylogenetic profile, which is matrix of the presence/absence of genes across the above bacteria, was created. Phylogenetic tree was built using the maximum likelihood method in MEGA5 [53] with 100 bootstraps. Tree topology was confirmed with the maximum likelihood method using PHYLIP v3.69. After searching, comparing and evaluating the T6SS in *A. avenae* subsp. *avenae* strain RS-1, the proteins of T6SS were analyzed for their association with the identified OM proteins regardless of *in vivo* or *in vitro*.

## Supporting Information

Figure S1 **Evolutionary relationship of T6SS of** ***Acidovorax avenae*** **subsp.** ***avenae*** **strain RS-1.** A distance tree (neighbor joining) was calculated from T6SS proteins sequences. Tree topology was confirmed with the maximum likelihood method using PHYLIP v3.69.
(TIF)

Table S1 **List of** ***in silico*** **predicated and LC/MS identified** ***in vitro*** **outer membrane protiens of** ***Acidovorax avenae*** **subsp.** ***avenae*** **strain RS-1 proteome.**
(XLS)

Table S2 **List of** ***in silico*** **predicted and LC/MS identified** ***in vivo*** **outer membrane protiens of** ***Acidovorax avenae*** **subsp.** ***avenae*** **strain RS-1 proteome.**
(XLS)

Table S3 **List of LC/MS identified shared outer membrane proteins under** ***in vivo*** **and** ***in vitro*** **of** ***Acidovorax avenae*** **subsp.** ***avenae*** **strain RS-1 proteome.**
(XLS)

Table S4 **List of LC-MS/MS identified unique outer membrane proteins under** ***in vitro*** **condition of** ***Acidovorax avenae*** **subsp.** ***avenae*** **strain RS-1 proteome.**
(XLS)

Table S5 **T6SS gene cluster including outer membrane proteins OmpA/MotB domain in** ***Acidovorax avenae*** **subsp.** ***avenae*** **strain RS-1 genome.**
(XLS)
