# Correction: Differential Expression of *In Vivo* and *In Vitro* Protein Profile of Outer Membrane of *Acidovorax avenae* Subsp. *avenae*

**DOI:** 10.1371/annotation/683735fe-858f-4f19-8ebd-ad840279ec88

**Published:** 2013-01-22

**Authors:** Muhammad Ibrahim, Yu Shi, Hui Qiu, Bin Li, Amara Jabeen, Liping Li, He Liu, Michael Kube, Guanlin Xie, Yanli Wang, Carlos Blondel, Carlos A. Santiviago, Inés Contreras, Guochang Sun

Carlos Blondel, Carlos Santiviago and Inés Contreras contributed the results under the section 'Genome Wide Analysis of T6SS Proteins' of the article, as well as Figure 3 and Table 2 in the article and should have been included as authors in this publication. The authors therefore wish to revise the author list to read as follows:

Muhammad Ibrahim, Yu Shi, Hui Qiu, Bin Li, Amara Jabeen, Liping Li, He Liu, Michael Kube, Guanlin Xie, Yanli Wang, Carlos Blondel, Carlos A Santiviago, Ines Contreras, Guochang Sun

Carlos Blondel, Carlos Santiviago and Inés Contreras are affiliated at the Departamento de Bioquímica y Biología Molecular, Facultad de Ciencias Químicas y Farmacéuticas, Universidad de Chile. Santiago, Chile. They have no competing interests to declare. Ines Contreras was supported by grant 1100092 from Fondo Nacional de Desarrollo Científico y Tecnológico (FONDECYT), Chile. Carlos J. Blondel was supported by Postdoctoral Fellowship 3120175 from FONDECYT, Chile. Carlos A. Santiviago was supported by grant 1110172 from FONDECYT, Chile. 

