# Supplementary figures and images for: Differential Expression of In Vivo and In Vitro Protein Profile of Outer Membrane of Acidovorax avenae Subsp. avenae
Source: PLoS One. 2012 Nov 15;7(11):e49657. doi: 10.1371/journal.pone.0049657 (PMC3499465; doi:10.1371/journal.pone.0049657)

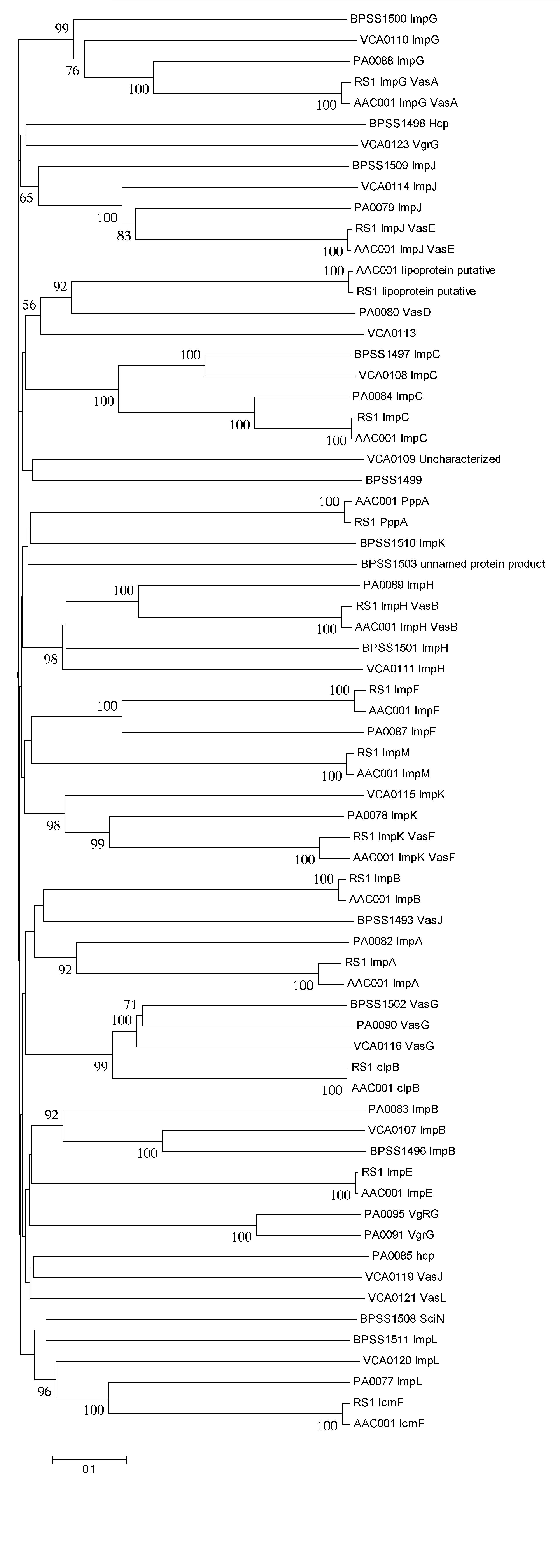

Supplement: Figure S1 — Evolutionary relationship of T6SS of Acidovorax avenae subsp. avenae strain RS-1. A distance tree (neighbor joining) was calculated from T6SS proteins sequences. Tree topology was confirmed with the maximum likelihood method using PHYLIP v3.69. (TIF) [file pone.0049657.s001.tif]
